# Chemical reaction enhanced graph learning for molecule representation

**DOI:** 10.1093/bioinformatics/btae558

**Published:** 2024-09-13

**Authors:** Anchen Li, Elena Casiraghi, Juho Rousu

**Affiliations:** Department of Computer Science, Aalto University, Espoo, 02150, Finland; Department of Computer Science, Aalto University, Espoo, 02150, Finland; AnacletoLab, Dipartimento di Informatica "Giovanni degli Antoni", University of Milan, Milan, 20133, Italy; Environmental Genomics and Systems Biology Division, Lawrence Berkeley National Laboratory, Berkeley, CA, 94720, United States; ELLIS, European Laboratory for Learning and Intelligent Systems, Milan Unit (University of Milan), Milan, 20133, Italy; Department of Computer Science, Aalto University, Espoo, 02150, Finland

## Abstract

**Motivation:**

Molecular representation learning (MRL) models molecules with low-dimensional vectors to support biological and chemical applications. Current methods primarily rely on intrinsic molecular information to learn molecular representations, but they often overlook effectively integrating domain knowledge into MRL.

**Results:**

In this article, we develop a reaction-enhanced graph learning (RXGL) framework for MRL, utilizing chemical reactions as domain knowledge. RXGL introduces dual graph learning modules to model molecule representation. One module employs graph convolutions on molecular graphs to capture molecule structures. The other module constructs a reaction-aware graph from chemical reactions and designs a novel graph attention network on this graph to integrate reaction-level relations into molecular modeling. To refine molecule representations, we design a reaction-based relation learning task, which considers the relations between the reactant and product sides in reactions. In addition, we introduce a cross-view contrastive task to strengthen the cooperative associations between molecular and reaction-aware graph learning. Experiment results show that our RXGL achieves strong performance in various downstream tasks, including product prediction, reaction classification, and molecular property prediction.

**Availability and implementation:**

The code is publicly available at https://github.com/coder-ACAC/RLM.

## 1 Introduction

Molecule representation learning (MRL) techniques are crucial for combining machine learning with biological and chemical sciences (Yi *et al.* 2022). MRL encodes molecules as low-dimensional vectors. These vectors retain molecule information, facilitating their use as features in downstream applications (e.g. product prediction, reaction classification, and molecular property prediction). A variety of MRL methods have been proposed, which roughly fall into the following two categories.

One school is SMILES-based methods (Fabian *et al.* 2020), which utilize SMILES strings as input and employ natural language models as their base architectures. However, they struggle with capturing molecule structures. The other school treats molecule topology as a graph, and models molecules with graph neural networks (GNNs) (Xu *et al.* 2021). Although GNN-based methods generally outperform SMILES-based ones, they typically focus on designing GNN architectures, neglecting the efficient integration of domain knowledge.

Recent studies (Wang *et al.* 2022a) use chemical reactions as domain knowledge for MRL. Typically, reactions are represented by equations, with reactants on the left side and products on the right (cf Definition 2 in Section Preliminaries). These methods first learn molecule embeddings from molecular graphs and then optimize embeddings by equating the sum of reactant embeddings with the sum of product embeddings for each reaction. Despite effectiveness, we argue that they face at least one of the following issues.

**Figure 1.**
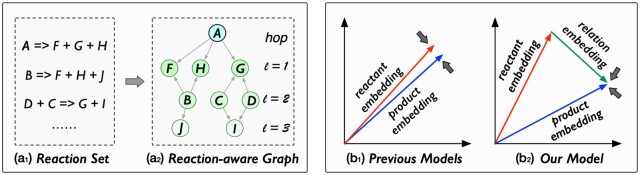
(a_1_) and (a_2_): An illustration of the reaction-aware graph. (b_1_) and (b_2_): Comparisons of previous models and our model in considering reactant–product relations of the chemical reaction.

Firstly, these reaction-based methods treat molecules as isolated data instances and rely solely on molecule structures for representation, which ignores the insights from molecule relations inherent in chemical reactions. For example, molecules involved in the same reaction (as reactants/products) may exhibit greater similarities and correlations with each other than with molecules from different reactions. To illustrate this reaction-related relation, we construct a reaction-aware graph (cf Definition 4 in Section Preliminaries) based on a reaction set, as shown in Fig. 1a. In this graph, nodes are molecules and edges denote molecule relations driven by reactions. For molecule *A*, its first-order neighbors (molecules *F*, *G*, and *H*) represent products that can be derived from *A* through reactions. Molecule *A*’s second-order neighbors (molecules *B*, *C*, and *D*) suggest a property/structure similarity with *A*, inferred from shared reaction products. Moreover, molecule *B* is likely more similar to *A* than *C* or *D*, as evidenced by a greater overlap in the reaction products. These analyses inspire us to consider the potential benefits of incorporating molecule relations from the reaction-aware graph into MRL.

Secondly, these methods ignore the transformation relation learning between reactants and products. Their assumption that the summed embeddings of reactants and products should be equal (as shown in Fig. 1b1) essentially reduces all reactions to an identity transformation, which oversimplifies the complexity of chemical processes. In reality, reactions involve various changes, such as the number of bonds (e.g. breaking old bonds and forming new ones) and energy variations (e.g. endotherms and exotherms) before and after the reaction. The assumption in current studies fails to model these changes.

Motivated by these gaps, we introduce a reaction-enhanced graph learning framework (RXGL) for MRL. In the molecule modeling stage, we design dual graph learning modules. The first module utilizes graph convolutions on molecular graphs to capture the structural information of molecules. The second module first involves a reaction-aware graph and then creates a GNN to extract reaction-level molecular relations for molecule feature learning. In the optimization stage, we introduce a reaction-based relation learning method that considers the relation between reactants and products in chemical reactions. Specifically, we employ a memory network (Miller *et al.* 2016) to learn a latent relation vector that connects reactant and product embeddings (as shown in Fig. 1b2). Through the delicate key and memory components in this network, the learned relation vectors could capture the hidden semantic correlations between the reactant and product in each reaction. Furthermore, we incorporate a cross-view contrastive learning task to enhance molecule representations in dual graph modeling. This task treats the molecular and reaction-aware graphs as distinct yet correlated views. By employing contrastive learning, we capture cooperative associations between views, thereby integrating their agreements into molecule representations.

**Figure 2.**
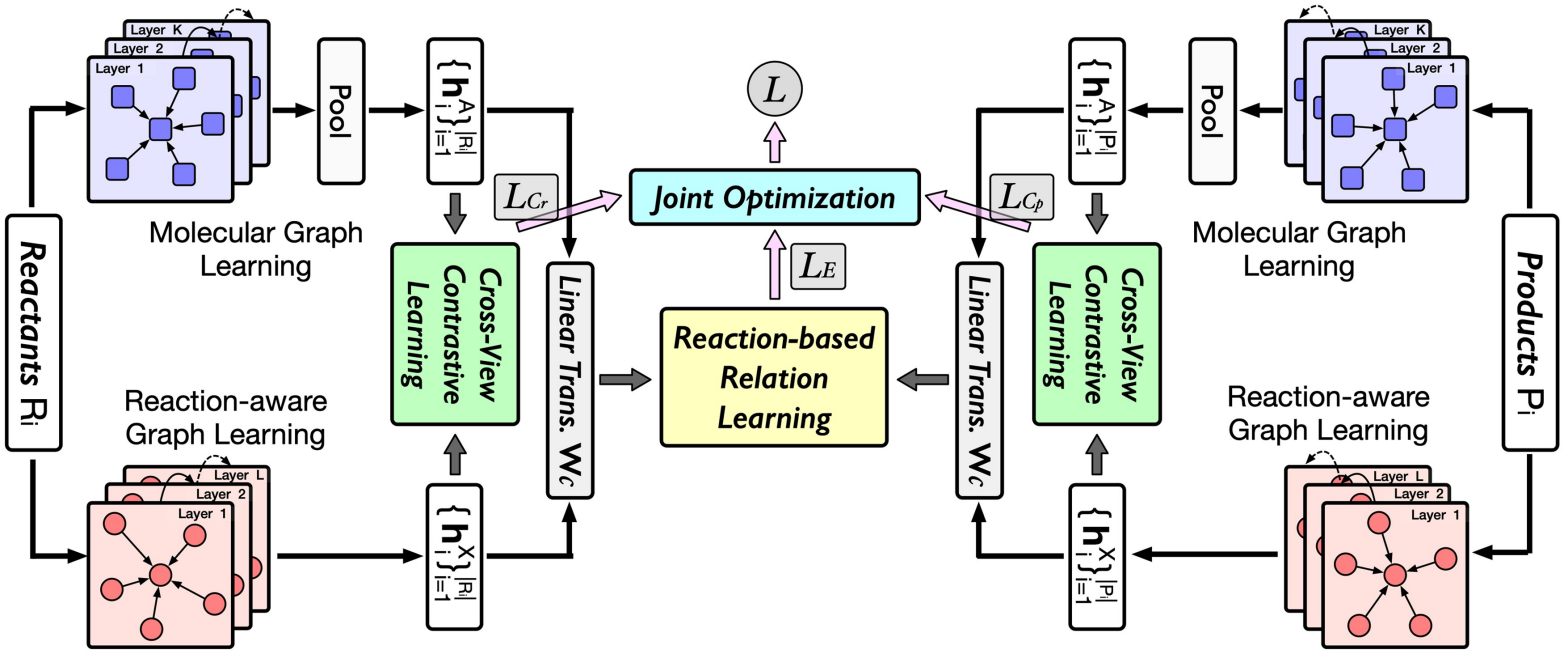
The framework of our RXGL method.

Our contributions are summarized as follows:

- We propose a GNN-based MRL framework RXGL, which introduces the chemical reaction-aware graph to assist in learning molecule representations.
- We devise a reaction-based relation modeling approach, which learns the relations between reactant and product sides in chemical reactions to guide MRL.
- We design a cross-view contrastive learning task to enhance the cooperative association between the molecular graph and reaction-aware graph modeling views.
- Experiment results show that molecule embeddings learned by our RXGL benefit various tasks, i.e. product prediction, reaction classification, and molecular property prediction.

## 2 Preliminaries

This section introduces notations and formulates the problem.

### 2.1 Notations

Our method RXGL uses the molecular graph and reaction-aware graph for molecule representation learning. We first introduce the definition of the molecular graph:

Definition 1. **Molecular Graph.** Given a molecule set M, each molecule m∈M has a graph structure $G_{m}=(V_{m},E_{m})$, which includes an atom node set $V_{m}$ and a bond edge set $E_{m}$. Each atom $a_{i}\in V_{m}$ is represented by a vector encoding its features, and bond $b_{i}\in E_{m}$ is represented by its bond type.

The reaction-aware graph is constructed based on chemical reactions. The chemical reaction is defined as follows:

Definition 2. **Chemical Reaction.** Consider a molecule set M and a reaction set X, a reaction $x_{i}\in X$ defines a transformation from a reactant set $R_{i}\subset M$ to a product set $P_{i}\subset M$, denoted as $x_{i}:R_{i}\to P_{i}$, where sets $R_{i}=\{r_{i_{1}},r_{i_{2}},\ldots \}$ and $P_{i}=\{p_{i_{1}},p_{i_{2}},\ldots \}$ represent the reactants and products involved in reaction $x_{i}$, respectively.

According to the definition of the chemical reaction, we define the reaction-level relation between molecules:

Definition 3. **Reaction-level Molecule Relation.** For a given reaction $x_{i}:$$R_{i}\to P_{i}$, a reaction-level molecule relation is defined between any reactant $r_{i_{a}}\in R_{i}$ and product $p_{i_{b}}\in P_{i}$.

Based on the reaction and reaction-level molecule relation, the reaction-aware graph is defined as follows:

Definition 4. **Reaction-aware Graph.** A reaction-aware graph is defined as $G_{x}=(V_{x},E_{x})$, where $V_{x}=R\cup P$ is the node set, and $E_{x}\subset R\times P$ is the edge set including reaction-level molecule relations between reactants R and products P.

Figure 1a shows an example of the reaction-aware graph. It is worth noticing that the construction of the reaction-aware graph draws inspiration from the metabolic networks (Wagner and Fell 2001) and chemical reaction networks (Wen *et al.* 2023) in synthetic biology. Its structural design facilitates the modeling of reaction-level molecule relations.

### 2.2 Problem formulation

Given the molecule $m_{i}$’s graph structure $G_{m_{i}}$, reaction-aware graph $G_{x}$ and a chemical reaction set X, we aim to learn $m_{i}$’s representation $h_{i}$. Then, learned molecule representation $h_{i}$ can be applied to various downstream tasks.

## 3 Reaction-enhanced graph learning (RXGL)

This section presents our RXGL method, as shown in Fig. 2. We first introduce dual graph learning modules to model molecule representations. Then, we propose a reaction-based relation learning task and a cross-view contrastive learning task to optimize molecule representations.

### 3.1 Molecule representation modeling

We introduce dual modules: a molecular graph learning module and a reaction-aware graph learning module. These modules are designed to integrate atom-level and reaction-level features into molecule representations, respectively.

#### 3.1.1 Molecular graph learning module

**Figure 3.**
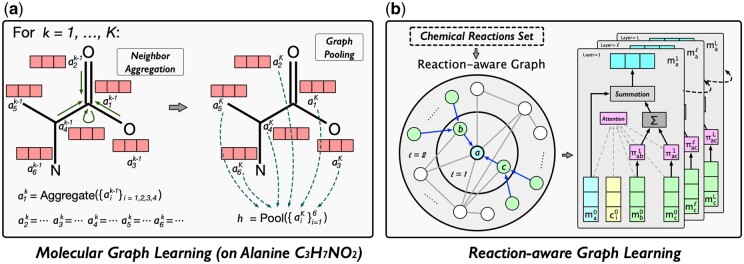
Molecular and reaction-aware graph learning.

This module first uses GNNs to model atoms and then utilizes pooling operations on the molecular graph to inject atom-level features into molecule representations, as shown in Fig. 3a. For molecule *m’*s graph structure $G_{m}=(V_{m},E_{m})$, we model representation $a_{i}^{k}$ of atom $a_{i}\in V_{m}$ in the *k*th GNN layer:

(1) $$a_{i}^{k}=\text{Aggregate}(a_{j}^{k-1}|a_{j}\in N_{i}\cup a_{i}),$$

where $N_{i}$ is the atoms connected to atom $a_{i}$ in graph $G_{m}$. The choice of Aggregate function is the key to designing GNN, leading to the proposal of various structures (Li *et al.* 2022, 2024). We utilize four common GNNs [i.e. GCN (Kipf and Welling 2017), GAT (Veličković *et al.* 2018), SAGE (Hamilton *et al.* 2017), and TAG (Du *et al.* 2017)] for this module. We introduce these GNNs in the Supplementary Materials.

To describe initial feature $a_{i}^{0}$ of atom $a_{i}$ in $G_{m}$, we use four types of atom properties: element type, charge, presence in an aromatic ring, and the number of attached hydrogen atoms. Each type of property is represented as a one-hot embedding, and four embeddings are concatenated as the initial atom feature. Note that we do not represent bond types with explicit feature vectors because the bond type could be inferred by the connected atom features (Wang *et al.* 2022a).

By stacking *K* GNN layers, a pooling function is used to generate atom-level molecule representation $h^{A}$, as follows:

(2) $$h^{A}=\text{Pool}(a_{i}^{K}|a_{i}\in V_{m})=\sum_{a_{i}\in V_{m}}a_{i}^{K}.$$

#### 3.1.2 Reaction-aware graph learning module

This module distills reaction-level relations from the reaction-aware graph to model molecule representations, as shown in Fig. 3b. We first construct a reaction-aware graph $G_{x}=(V_{x},E_{x})$. To describe initial feature $m_{i}$ of molecule $m_{i}\in V_{x}$ in $G_{x}$, we use the molecule functional group information. We first use a one-hot embedding to denote each functional group, and then the molecule is represented by the summation of its functional group embeddings. We consider 39 functional groups, which can be found in the Supplementary Materials.

We design a GNN to model molecule representations in $G_{x}$. Guided by chemical reactions, it employs an attention mechanism for aggregating neighbor information. Specifically, for molecule $m_{i}$ in reaction x:R→P (i.e. $m_{i}\in R\cup P$), its neighbor aggregation in the *l*th layer is defined as:

(3) $$m_{i}^{l}=m_{i}^{l-1}+\sum_{j\in N_{i}}\pi_{ij}^{l}m_{j}^{l-1},$$

where $m_{i}^{l}$ is $m_{i}$’s representation ($m_{i}^{0}=m_{i}$), $\pi_{ij}$ denotes the attention score between $m_{i}$ and its neighbor $m_{j}\in N_{i}$ in $G_{x}$. To enhance computational efficiency, we sample a fixed-size neighbor subset from $N_{i}$ for each molecule. The size of the subset is a predefined constant *Q*.

The attention score $\pi_{ij}$ is defined as follows:

(4) $$\pi_{ij}^{l}=\frac{\;\text{exp}(W_{a}^{l}(m_{i}^{l-1}⊙m_{j}^{l-1}⊙c^{l-1}))}{\sum_{k\in N_{i}}\;\text{exp}\;(W_{a}^{l}(m_{i}^{l-1}⊙m_{k}^{l-1}⊙c^{l-1}))},$$

where ⊙ denotes the element-wise product between vectors, $W_{a}$ is the weight matrix, and c is the contextual information for molecule $m_{i}$ in chemical reaction x:R→P. The reaction context c is defined as $c^{l}=\sum_{m_{u}\in R\cup P}m_{u}^{l}$. Our attention mechanism considers the reaction context, allowing for the control in neighbor message passing.

After stacking *L* layers of GNN, we utilize representation $m_{i}^{L}$ as molecule $m_{i}$’s reaction-level representation $h_{i}^{X}$.

### 3.2 Molecule representation optimization

For each molecule $m_{i}$, we derive its two representations (i.e. $h_{i}^{A}$ and $h_{i}^{X}$) from dual graph learning. To model $m_{i}$’s final representation $h_{i}$, we use a linear transformation with weight $W_{c}$ and concatenation operation ||, as $h_{i}=W_{c}(h_{i}^{A}||h_{i}^{X})$.

To further optimize these molecular representations, we introduce a reaction-based relation learning task and a cross-view contrastive learning task. The former models the relation between reactants and products in each reaction. The latter is designed to enhance the cooperative association between molecular and reaction-aware graph learning.

#### 3.2.1 Reaction-based relation learning task

This task models reactant and product pairs using the relation vector, as shown in Fig. 1b2. Given a chemical reaction $x_{i}:R_{i}\to P_{i}$, we assume that equation $x_{R_{i}}+e_{R_{i}\to P_{i}}=x_{P_{i}}$ holds, where $x_{R_{i}}$, $x_{P_{i}}$, and $e_{R_{i}\to P_{i}}$ are the representations of reactant $R_{i}$, product $P_{i}$, and relation from $R_{i}$ to $P_{i}$, respectively. First, we use the summation operation to define $x_{R_{i}}$ and $x_{P_{i}}$, as:

(5) $$x_{R_{i}}=\sum_{m_{j}\in R_{i}}h_{j},\;\;\;\;\;\;x_{P_{i}}=\sum_{m_{k}\in P_{i}}h_{k}.$$

**Figure 4.**
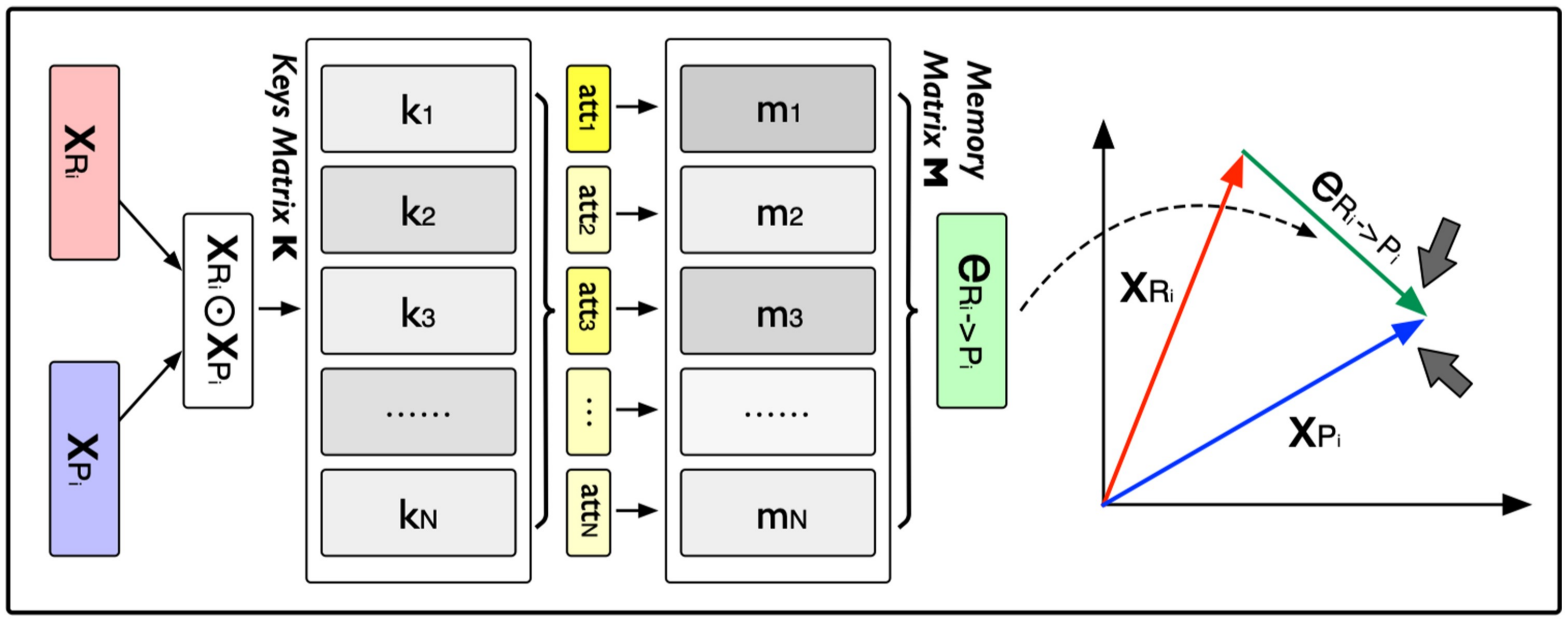
Reaction-based relation learning.

To model the relation vector $e_{R_{i}\to P_{i}}$ of reaction $x_{i}$, we introduce a memory network (as shown in Fig. 4) which takes a reactant–product pair $\left(x_{R_{i}},x_{P_{i}}\right)$ as input, and outputs the relation vector $e_{R_{i}\to P_{i}}$ of equal dimension as $x_{R_{i}}$ and $x_{P_{i}}$.

Let $R^{d}$ be the dimension of $x_{R_{i}}$ and $x_{P_{i}}$. The network first introduces a key matrix $K\in R^{N\times d}$ to calculate an attention vector $\mathrm{att}\in R^{N}$. The element $att_{n}\in \mathrm{att}$ is defined as:

(6) $$att_{n}=\frac{\;\text{exp}({\left(x_{R_{i}}⊙x_{P_{i}}\right)}^{⊤}k_{n})}{\sum_{m=1}^{N}\;\text{exp}\;({\left(x_{R_{i}}⊙x_{P_{i}}\right)}^{⊤}k_{m})},$$

where $k\in R^{d}$ is the vector element of matrix K.

Next, we introduce a memory matrix $M\in R^{N\times d}$ to generate the relation vector $e_{R_{i}\to P_{i}}$, as follows:

(7) $$e_{R_{i}\to P_{i}}=\sum_{n=1}^{N}att_{n}\cdot m_{n},$$

where $m\in R^{d}$ is the vector element of matrix M.

The relation vector $e_{R_{i}\to P_{i}}$ is a weighted representation of **M**. Intuitively, the memory matrix **M** can be interpreted as a store of conceptual building blocks that can be used to describe the relations between reactants and products.

After the relation modeling, we define the following score function for reactant–product pair $R_{i}$ and $P_{i}$ of reaction $x_{i}$:

(8) $$S(R_{i},P_{i})=||x_{R_{i}}+e_{R_{i}\to P_{i}}-x_{P_{i}}|{|}_{2}.$$

For optimization, we utilize a contrastive learning method similar to (Radford *et al.* 2021). In a minibatch of reaction data $X_{B}$, we identify matched reactant–product pairs as positive pairs, aiming to minimize their embedding differences, while unmatched pairs are considered negative pairs, with an objective to maximize their embedding discrepancies. We use the margin-based loss function (Bordes *et al.* 2013), as follows:

(9) $$\begin{array}{c} L_{E}=\frac{1}{\left|X_{B}\right|}\sum_{x_{i}\in X_{B}}S(R_{i},P_{i})+\\ \begin{array}{c} \;\;\;\;\;\frac{1}{\left|X_{B}{|}^{2}-|X_{B}\right|}\sum_{x_{i}\in X_{B}}\sum_{x_{j}\in X_{B}}\text{max}(\gamma -S(R_{i},P_{j}),0), \end{array} \end{array}$$

where $x_{i}\neq x_{j}$ and γ>0 is a margin hyperparameter.

#### 3.2.2 Cross-view contrastive learning task

This task enhances representations of molecules by aligning outputs from molecular and reaction-aware graph learning modules. We consider different views of the same reactant or product as positive pairs, while views of different reactants or products form negative pairs. This method enables our model to learn distinct representations by contrasting these positive and negative instances. Since contrastive tasks of reactants and products are similar, we illustrate the reactant side task.

Specifically, for a reaction $x_{i}$, we first generate its two reactant representations $x_{R_{i}}^{A}$ and $x_{R_{i}}^{X}$ from the molecular and reaction-aware graph learning modules, respectively, as:

(10) $$x_{R_{i}}^{A}=\sum_{m_{j}\in R_{i}}h_{j}^{A},\;\;\;\;\;\;x_{R_{i}}^{X}=\sum_{m_{k}\in R_{i}}h_{k}^{X}.$$

Then, we enforce the separation of different reactant representations and align those that are identical. To achieve this, we employ InfoNCE (Oord *et al.* 2018) to define the cross-view contrastive loss for reactants as follows:

(11) $$L_{C_{r}}=\sum_{x_{i}\in X_{B}}-\text{log}\;\frac{\;\text{exp}(c(x_{R_{i}}^{A},x_{R_{i}}^{X})/\tau )}{\sum_{x_{j}\in X_{B}}\;\text{exp}\;(c(x_{R_{i}}^{A},x_{R_{j}}^{X})/\tau )},$$

where $X_{B}$ denotes a minibatch of reaction data, c(·,·) is the cosine similarity, and τ is a hyper-parameter for the softmax function. Here we use a minibatch-based contrastive strategy for its efficiency in terms of both time and memory.

Similarly, we calculate contrastive loss $L_{C_{p}}$ for the product side. Combining two losses, we formulate the objective function of our cross-view contrastive task as $L_{C}=L_{C_{r}}+L_{C_{p}}$.

#### 3.2.3 Optimization function

Our RXGL is trained using a weighted sum of losses from the reaction relation learning and cross-view contrastive tasks.

(12) $$L=L_{E}+\alpha L_{C}+\lambda ||\Theta |{|}_{F}^{2},$$

where Θ is model parameters, and α and λ adjust the cross-view contrastive task and L2-regularization, respectively.

We use the minibatch-based negative sampling strategy for two tasks, offering advantages over the traditional approach (Mikolov *et al.* 2013). First, it requires no extra memory to store negative samples. Second, negative samples are refreshed each epoch due to the shuffling of training instances, saving the time for manual re-sampling.

## 4 Experiments

In this section, we conduct extensive experiments to show the effectiveness of our approach RXGL in several downstream tasks, including product prediction, reaction classification, and molecular property prediction.

### 4.1 Product prediction

Reaction product prediction is a fundamental problem in the biology and chemistry field, which aims to predict the products of a reaction based on the reactants.

**Dataset**. We conduct experiments on two biochemistry benchmark datasets: USPTO-15K (Jin *et al.* 2017), containing 15 000 reactions, and USPTO-50K (Liu *et al.* 2024), comprising 50 000 reactions. Each dataset was divided into training, validation, and test sets using an 8:1:1 ratio.

**Evaluation protocol**. Following (Wang *et al.* 2022a), we formulate the product prediction as a ranking task. Let $X_{\mathrm{test}}$ and $P_{\mathrm{test}}$ be the sets of reactions and products in the test set. For each reaction $x_{i}:R_{i}\to P_{i}$ in $X_{\mathrm{test}}$, we rank all products $P_{j}\in P_{\mathrm{test}}$ based on the score function $S(R_{i},P_{j})$ [i.e. Equation (8)]. Then, the ranking of the ground-truth candidate can be used for the evaluation. Our evaluation metrics are MRR (mean reciprocal rank) and Hit@1 (hit ratio at a cut-off value of 1). We conduct each experiment five times, reporting both the mean and standard deviation on the test set, with the results selected based on the best MRR in the validation set.

**Baselines**. Our RXGL is compared with Mol2vec (Jaeger *et al.* 2018), MolBERT (Fabian *et al.* 2020), and MolR (Wang *et al.* 2022a). For Mol2vec and MolBERT, we employ their pre-trained models to generate embeddings for reactants and products. Then, aligning with MolR, the scoring function is defined through an inner product. For MolR and our RXGL, we use four GNNs (i.e. GCN, GAT, SAGE, and TAG) as encoders for the molecular graph. We present the details of these encoders in the Supplementary Materials.

**Hyperparameter settings**. We train our model RXGL for 50 epochs with a batch size of 1024, using Adam optimizer with a learning rate of $10^{-4}$. The number of GNN layers in molecular and reaction-aware graph learning is set to 2 and 1, respectively, and the output dimension of all layers is 256. In addition, neighbor sampling size *Q*, memory slice size *N*, margin γ, temperature τ, and balancing factor α are set to 10, 10, 4, 0.1, and $10^{-3}$, respectively. The result of the key hyperparameter sensitivity is reported in Section 4.4.

**Performance comparison**. The results are shown in Table 1. We find that our RXGL performs best. To be specific, RXGL-SAGE achieves 14.1% average MRR gain and 16.3% average Hit@1 gain over baselines on the USPTO-15K dataset, showing the effectiveness of RXGL in the product prediction.

**Table 1.** Results of the product prediction task on the two datasets.^a^

Methods	USPTO-15K		USPTO-50K	
	MRR	Hit@1	MRR	Hit@1
Mol2vec	0.519	0.468	0.835	0.801
MolBERT	0.790	0.734	0.913	0.874
MolR-GCN	0.883${}_{\left(0.005\right)}$	0.847${}_{\left(0.007\right)}$	0.958${}_{\left(0.003\right)}$	0.944${}_{\left(0.006\right)}$
MolR-GAT	0.881${}_{\left(0.003\right)}$	0.846${}_{\left(0.002\right)}$	0.952${}_{\left(0.002\right)}$	0.931${}_{\left(0.004\right)}$
MolR-SAGE	0.932${}_{\left(0.006\right)}$	0.905${}_{\left(0.003\right)}$	0.972${}_{\left(0.005\right)}$	0.960${}_{\left(0.005\right)}$
MolR-TAG	0.925${}_{\left(0.005\right)}$	0.898${}_{\left(0.004\right)}$	0.974${}_{\left(0.010\right)}$	0.965${}_{\left(0.009\right)}$
RXGL-GCN	0.927${}_{\left(0.003\right)}$	0.899${}_{\left(0.005\right)}$	0.965${}_{\left(0.007\right)}$	0.954${}_{\left(0.002\right)}$
RXGL-GAT	0.925${}_{\left(0.007\right)}$	0.894${}_{\left(0.006\right)}$	0.967${}_{\left(0.009\right)}$	0.958${}_{\left(0.011\right)}$
RXGL-SAGE	**0.956** ${}_{\left(0.004\right)}$	**0.936** ${}_{\left(0.007\right)}$	**0.982** ${}_{\left(0.005\right)}$	0.973${}_{\left(0.003\right)}$
RXGL-TAG	0.941${}_{\left(0.006\right)}$	0.919${}_{\left(0.009\right)}$	0.979${}_{\left(0.004\right)}$	**0.974** ${}_{\left(0.009\right)}$

a The numbers in brackets are the standard deviations.

Bold values denote the best values of all methods.

### 4.2 Reaction classification

Reaction classification aims to predict the class of reactions.

**Dataset**. Our RXGL is evaluated using two datasets, i.e. Schneider and USPTO-MTL. Specifically, Schneider (Schneider *et al.* 2015) comprises 38 800 reactions across 46 classes, and USPTO-MTL (Lu and Zhang 2022) includes 143 535 reactions across 1000 classes. Each dataset is randomly split into training, validation, and test sets in an 8:1:1 ratio.

**Baselines and experiment settings**. Similar to product prediction, we compare our RXGL with Mol2vec, MolBERT, and MolR. We employ our model pre-trained on the USPTO-50k dataset to process all datasets, which includes generating embeddings for reactants and products in each reaction and concatenating them to create a unified reaction feature. For prediction, we use an MLP as the decoder. Accuracy and Recall are used as evaluation metrics. For each experiment, we perform five times and report the mean and standard deviation of the results on the test set.

**Performance comparison**. Table 2 shows the results of the reaction classification task. We find that our RXGL outperforms baselines. For example, RXGL-SAGE achieves an average Accuracy increase of 4.0% on Schneider dataset. These results highlight RXGL’s efficacy in the reaction classification task, and suggest that the molecule representations it learns are effectively transferable to the downstream task.

**Table 2.** Results of the reaction classification task.^a^

Methods	Schneider		USPTO-MTL	
	Accuracy	Recall	Accuracy	Recall
Mol2vec	0.856${}_{\left(0.012\right)}$	0.850${}_{\left(0.009\right)}$	0.751${}_{\left(0.016\right)}$	0.629${}_{\left(0.014\right)}$
MolBERT	0.849${}_{\left(0.014\right)}$	0.847${}_{\left(0.012\right)}$	0.738${}_{\left(0.009\right)}$	0.583${}_{\left(0.008\right)}$
MolR-GCN	0.879${}_{\left(0.013\right)}$	0.882${}_{\left(0.013\right)}$	0.853${}_{\left(0.020\right)}$	0.813${}_{\left(0.015\right)}$
MolR-GAT	0.870${}_{\left(0.024\right)}$	0.873${}_{\left(0.021\right)}$	0.862${}_{\left(0.017\right)}$	0.834${}_{\left(0.014\right)}$
MolR-SAGE	0.882${}_{\left(0.030\right)}$	0.881${}_{\left(0.027\right)}$	0.874${}_{\left(0.023\right)}$	0.839${}_{\left(0.026\right)}$
MolR-TAG	0.891${}_{\left(0.025\right)}$	0.895${}_{\left(0.026\right)}$	0.888${}_{\left(0.024\right)}$	0.852${}_{\left(0.024\right)}$
RXGL-GCN	0.887${}_{\left(0.016\right)}$	0.889${}_{\left(0.017\right)}$	0.875${}_{\left(0.018\right)}$	0.849${}_{\left(0.015\right)}$
RXGL-GAT	0.872${}_{\left(0.026\right)}$	0.874${}_{\left(0.020\right)}$	0.873${}_{\left(0.019\right)}$	0.836${}_{\left(0.016\right)}$
RXGL-SAGE	**0.907** ${}_{\left(0.029\right)}$	**0.906** ${}_{\left(0.025\right)}$	0.889${}_{\left(0.014\right)}$	0.858${}_{\left(0.018\right)}$
RXGL-TAG	0.899${}_{\left(0.033\right)}$	0.901${}_{\left(0.031\right)}$	**0.895** ${}_{\left(0.019\right)}$	**0.867** ${}_{\left(0.018\right)}$

a The numbers in brackets are the standard deviations.

Bold values denote the best values of all methods.

### 4.3 Molecular property prediction

This task is to predict labels of given molecules, which is a classical task to evaluate learned molecule representations.

**Dataset**. We evaluate our RXGL on four datasets (Wu *et al.* 2018): BBBP, BACE, Tox21, and ClinTox. Each dataset contains molecule SMILES as well as labels indicating the property. Readers can refer to (Wu *et al.* 2018) for a detailed introduction to these four public datasets.

**Baselines**. We compare our RXGL (i.e. variant RXGL-GCN) with the following four class methods: (i) SMILES-based methods: ChemBERTa (Chithrananda *et al.* 2020) and MolBERT (Fabian *et al.* 2020); (ii) Fingerprint-based methods: Mol2vec (Jaeger *et al.* 2018), ECFP4 (Rogers and Hahn 2010), GraphConv (Duvenaud *et al.* 2015), Weave (Kearnes *et al.*, 2016), D-MPNN (Yang *et al.* 2019), CDDD (Winter *et al.* 2019); (iii) GNN-based methods: GraphCL (You *et al.* 2020), GraphLoG (Xu *et al.* 2021), EdgePred (Hamilton *et al.* 2017), AttrMask (Hu *et al.* 2019), GPT-GNN (Hu *et al.* 2020), InfoGraph (Sun *et al.* 2020), ContextPred (Hu *et al.* 2019), G-Motif (Rong *et al.* 2020), JOAO (You *et al.* 2021), and GraphMVP (Liu *et al.* 2022); (iv) Reaction-enhanced methods: MolR (Wang *et al.* 2022a) and ReaKE (Wang *et al.* 2022b).

**Experiment settings**. We split all datasets into training, validation, and test sets in an 8:1:1 ratio, using two split types: Random and scaffold. Our model pre-trained on USPTO-50K datasets is used to generate molecule embeddings, which are formed by concatenating outputs from the molecular graph learning module with the sum of molecule functional group embeddings. Then, we input the molecule embeddings along with their labels into a logistic regression model. We use the AUC (area under the curve) as our evaluation metric. Each experiment is conducted five times, and we report the mean and standard deviation of the results on the test set.

**Performance comparison**. Different baselines leverage different strategies (i.e. random or scaffold) to split datasets. We compare our model with baselines by adopting their selected dataset splitting. The AUC results for molecular property prediction are presented in Tables 3 and 4. The baseline results are taken from the literature. Our RXGL shows strong performance across four datasets, e.g. RXGL exhibits average AUC gains of 17.8%, 7.2%, 7.9%, and 11.3% on four datasets under random splitting, showing that learned molecule representations effectively transfer to molecule-related tasks.

**Table 3.** Molecular property prediction results (split type: *random split*).^a^

Methods	BBBP	BACE	Tox21	ClinTox
ChemBERTa^★^	0.643	–	0.728	0.733
MolBERT^★^	0.762${}_{\left(0.000\right)}$	0.866${}_{\left(0.000\right)}$	–	–
Mol2vec^★^	0.872${}_{\left(0.021\right)}$	0.862${}_{\left(0.027\right)}$	0.803${}_{\left(0.041\right)}$	0.841${}_{\left(0.062\right)}$
ECFP4^★^	0.729	0.867	0.822	0.799
GraphConv^★^	0.690	0.783	0.829	0.807
Weave^★^	0.671	0.806	0.820	0.832
D-MPNN^★^	0.708	–	0.688	0.906
CDDD^★^	0.761${}_{\left(0.000\right)}$	0.833${}_{\left(0.000\right)}$	–	–
GraphCL^★^	0.695${}_{\left(0.005\right)}$	0.782${}_{\left(0.012\right)}$	0.754${}_{\left(0.009\right)}$	0.701${}_{\left(0.019\right)}$
GraphLoG^★^	0.725${}_{\left(0.008\right)}$	0.835${}_{\left(0.012\right)}$	0.757${}_{\left(0.005\right)}$	0.767${}_{\left(0.033\right)}$
MolR^★^	0.895${}_{\left(0.031\right)}$	0.875${}_{\left(0.023\right)}$	0.820${}_{\left(0.028\right)}$	0.913${}_{\left(0.043\right)}$
ReaKE${}^{♠}$	–	0.898	0.824	0.874
RXGL	**0.901** ${}_{\left(0.024\right)}$	**0.906** ${}_{\left(0.017\right)}$	**0.852** ${}_{\left(0.026\right)}$	**0.921** ${}_{\left(0.030\right)}$

a The numbers in brackets are the standard deviations. The results of symbols ★ and ♠ are taken from MolR and ReaKE.

Bold values denote the best values of all methods.

**Table 4.** Property prediction results (split type: *scaffold split*).^a^

Methods	BBBP	BACE	Tox21	ClinTox
EdgePred^★^	0.645${}_{\left(0.031\right)}$	0.646${}_{\left(0.047\right)}$	0.745${}_{\left(0.004\right)}$	0.558${}_{\left(0.062\right)}$
AttrMask^★^	0.702${}_{\left(0.005\right)}$	0.772${}_{\left(0.014\right)}$	0.742${}_{\left(0.008\right)}$	0.686${}_{\left(0.096\right)}$
GPT-GNN^★^	0.645${}_{\left(0.011\right)}$	0.776${}_{\left(0.005\right)}$	0.753${}_{\left(0.005\right)}$	0.578${}_{\left(0.031\right)}$
InfoGraph^★^	0.692${}_{\left(0.008\right)}$	0.739${}_{\left(0.025\right)}$	0.730${}_{\left(0.007\right)}$	0.751${}_{\left(0.050\right)}$
ContextPred^★^	0.712${}_{\left(0.009\right)}$	0.786${}_{\left(0.014\right)}$	0.733${}_{\left(0.005\right)}$	0.737${}_{\left(0.040\right)}$
G-Contextual^★^	0.703${}_{\left(0.016\right)}$	0.792${}_{\left(0.003\right)}$	0.752${}_{\left(0.003\right)}$	0.599${}_{\left(0.082\right)}$
G-Motif^★^	0.664${}_{\left(0.034\right)}$	0.734${}_{\left(0.040\right)}$	0.732${}_{\left(0.008\right)}$	0.778${}_{\left(0.020\right)}$
JOAO^★^	0.660${}_{\left(0.006\right)}$	0.729${}_{\left(0.020\right)}$	0.744${}_{\left(0.007\right)}$	0.663${}_{\left(0.039\right)}$
GraphMVP^★^	0.724${}_{\left(0.016\right)}$	0.812${}_{\left(0.009\right)}$	**0.744** ${}_{\left(0.002\right)}$	0.775${}_{\left(0.042\right)}$
MolR${}^{♠}$	–	0.774	0.670	0.830
ReaKE${}^{♠}$	–	0.781	0.713	0.862
RXGL	**0.729** ${}_{\left(0.015\right)}$	**0.825** ${}_{\left(0.006\right)}$	0.736${}_{\left(0.003\right)}$	**0.912** ${}_{\left(0.011\right)}$

a The numbers in brackets are the standard deviations. The results of symbols ^★^ and ♠ are taken from GraphMVP and ReaKE.

Bold values denote the best values of all methods.

### 4.4 Hyper-parameter study

**Figure 5.**
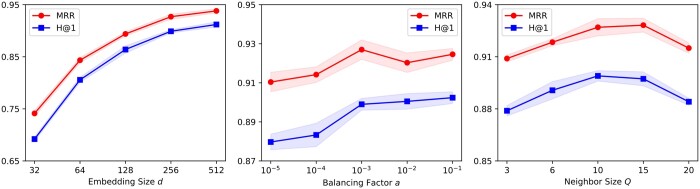
Hyper-parameter study on the USPTO-15K dataset.

We study three hyper-parameters on RXGL-GCN: Embedding size (*d*), balancing factor (α), and neighbor sampling size (*Q*), as shown in Fig. 5. We find: (i) A larger *d* typically enhances performance by encoding more information, yet an excessively large *d* increases the parameter size. We suggest setting *d* at 256 to balance performance and storage requirements. (ii) Nice performance is achieved when α is set to 0.001. We hypothesize that a small α might inadequately reinforce the cooperative associations between molecular and reaction-aware graph learning. (iii) The rank accuracy improves with increasing *Q* but eventually decreases. This decline may be due to the introduction of irrelevant information from too many neighbors.

### 4.5 Embedding analysis

This subsection studies the learned molecule embeddings and reaction-based relation embeddings [i.e. Equation (7)].

#### 4.5.1 Molecule embedding analysis

**Figure 6.**
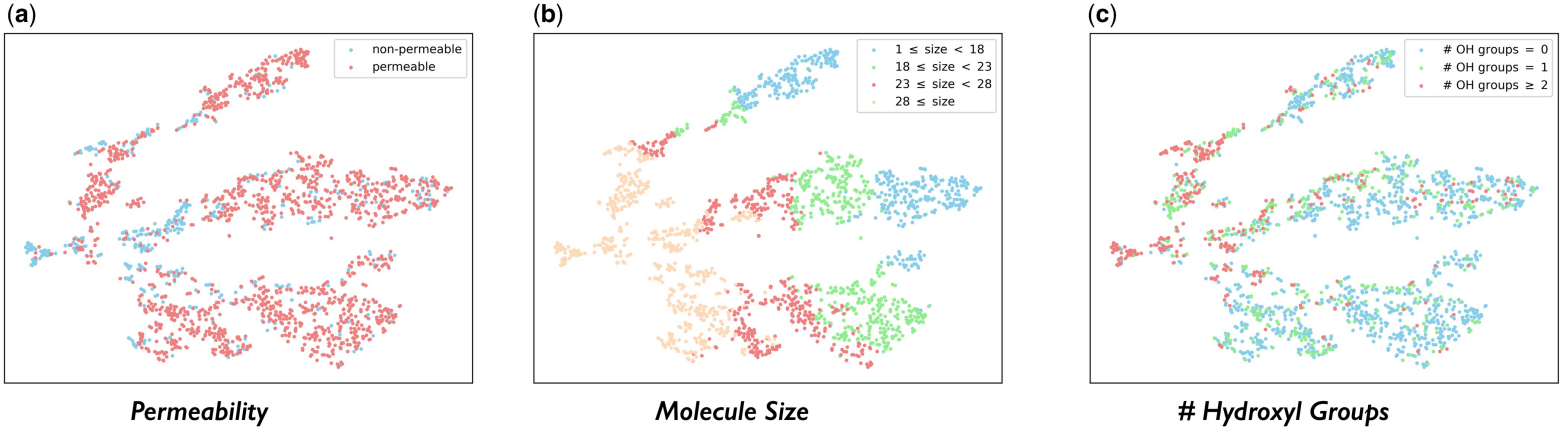
Visualized molecule embeddings on the BBBP dataset.

We first use RXGL-GCN to generate embeddings of molecules in the BBBP dataset and visualize them utilizing the t-SNE method (Van der Maaten and Hinton 2008). Specifically, we select the molecules’ permeability property, molecule size (i.e. the number of atoms), and 39 functional group properties. These 39 functional groups are provided by RDKit (Landrum 2013). Due to the space limitation, we present visualizations in the main text for the permeability, the molecule size, and the hydroxyl functional group properties. More detailed information about these functional groups and remaining experimental results can be found in Supplementary Materials. From visualization results, we find that: Figure 6a illustrates molecules colored according to their permeability properties. Several distinct clusters of nonpermeable molecules are observed. Figure 6b shows molecules colored by size. The embedding space distinctly segregates small molecules (located in the right region) from large molecules (located in the left region). Figure 6c shows molecules colored based on the number of the hydroxyl functional groups (i.e. ‘–OH’). Molecules with a higher number of hydroxyl groups are mainly on the left side of the embedding space, while those without this group are primarily found on the right side. In summary, these results indicate that the molecular embeddings generated by our RXGL framework exhibit a certain degree of correlation with the aforementioned three molecular properties.

#### 4.5.2 Reaction-based relation embedding analysis

In our RXGL, we learn relation embeddings between reactants and products in chemical reactions. This subsection examines the meaningfulness of these relation embeddings.

**Figure 7.**
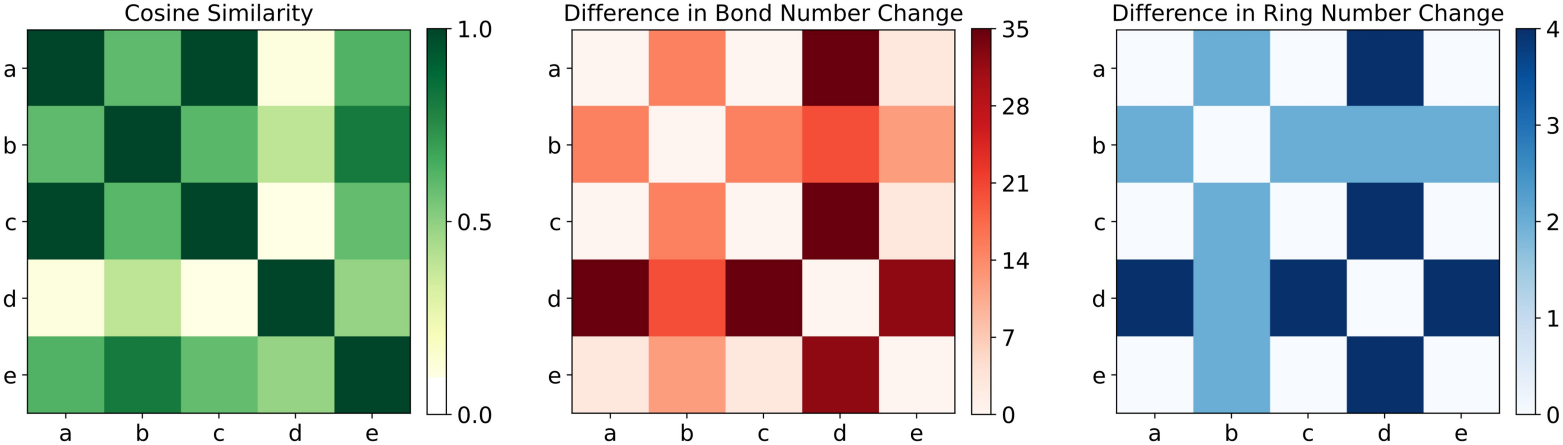
Examples of relation embedding comparison.

A key feature of chemical reactions is the change of reactants into products. For example, this process involves breaking old bonds and forming new ones, resulting in a change in the number of bonds before and after the reaction. Intuitively, reactions with similar changes yield similar relation embeddings. To analyze this, we randomly select five reactions (labeled *a*, *b*, *c*, *d*, and *e*) from the test set of USPTO-15K dataset and compute the cosine similarity of their relation embeddings, as shown in the left subfigure of Fig. 7. Take reaction *a* as an example, we observe a high similarity between *a* and *c* and a low similarity between *a* and *d*. We characterize chemical reaction changes by the difference in bond number ($N_{B}$) and ring number ($N_{R}$) in reactants and products. The middle and right subfigures of Fig. 7 show differences between these five chemical reactions in terms of bond number changes and ring number changes. For example, a small square in the middle subfigure represents $\left|N_{B}(i)-N_{B}(j)\right|$, which means the differences between reactions *i* and *j* in terms of bond number changes. From the results, we find that the high cosine similarity between reactions *a* and *c* is likely due to their similar bond and ring number changes. Conversely, the low similarity between reactions *a* and *d* can be attributed to their significant differences in bond and ring number changes.

**Figure 8.**
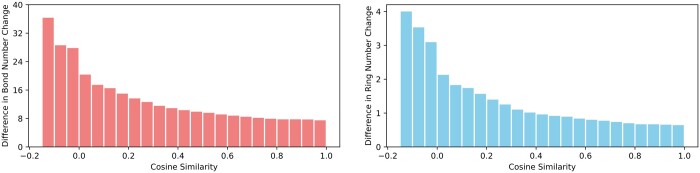
Macro-analysis on the USPTO-15K dataset.

We further conduct the macro-analysis. We calculate the cosine similarity between every reaction pair in the test set and examine the differences in bond number and ring number changes between each pair. We show the results on the USPTO-15K dataset in Fig. 8, where the *y*-axis represents the average change within a certain similarity interval. We find that reaction pairs with high cosine similarities tend to exhibit small differences in bond and ring number changes, and vice versa. The above observation underscores the significance of our learned relation embeddings, suggesting their capability to model the changes occurring in chemical reactions.

## 5 Conclusion and future work

This article proposes RXGL that uses reactions as domain knowledge in MRL. RXGL integrates molecular and reaction-aware graph learning modules to model molecule representations. Also, we enhance molecule representations using a reaction-based relation learning task and a cross-view contrastive task. Experiment results show that RXGL achieves strong performance across a range of downstream tasks. We believe that the insights of this study will provide valuable guidance for future research exploring the utilization of reactions in MRL. For future work, we plan to consider the incorporation of stereochemical information into RXGL, which will enhance the accuracy and applicability of molecule representations.

## Supplementary Material

btae558_Supplementary_Data

## Data Availability

No new data were generated or analyzed in support of this research.
