# Supplementary material for: Chemical reaction enhanced graph learning for molecule representation
Source: Bioinformatics. 2024 Sep 13;40(10):btae558. doi: 10.1093/bioinformatics/btae558 (PMC11639130; doi:10.1093/bioinformatics/btae558)
Supplement: btae558_Supplementary_Data [file btae558_supplementary_data.pdf]

Supplementary data: Chemical Reaction Enhanced Graph Learning for Molecule Representation

Anchen Li[1], Elena Casiraghi[1,2,3,4], and Juho Rousu[1]
1.Department of Computer Science, Aalto University, Finland; 2.AnacletoLab, Dipartimento di Informatica, University of Milan, Italy; 3.Environmental Genomics and Systems Biology Division, Lawrence Berkeley National Laboratory, Berkeley, CA, USA; 4.ELLIS, European Laboratory for Learning and Intelligent Systems, Milan Unit, Italy
anchen.li@aalto.fi

In the supplementary data, we introduce some details which have been omitted in the paper.

A. GNNs for Molecular Graph Learning

In molecular graph learning, we utilize graph neural networks (GNNs) to model the molecule structure. Specifically, for a molecular graph $\mathcal{G}_m = (\mathcal{V}_m, \mathcal{E}_m)$, we model representation $\mathbf{a}_i^k$ of atom $a_i \in \mathcal{V}_m$ in the k-th GNN layer, as:

$$\mathbf{a}_i^k = \text{Aggregate}(\mathbf{a}_j^{k-1} | a_j \in \mathcal{N}_i \cup a_i), \tag{1}$$

where $\mathcal{N}_i$ is the atom neighbor set connected to atom a_i in graph $\mathcal{G}_m$.

The choice of the Aggregate function is the key to designing GNN. In this paper, we choose four GNNs (i.e., GCN [1], GAT [2], SAGE [3], and TAG[4]).

These GNNs are defined as follows:

- **GCN** (graph convolutional networks [1]). In GCN, the Aggregate function is defined as:

$$\mathbf{a}_i^k = \sigma\Big(\sum\nolimits_{j \in \mathcal{N}_i \cup a_i} \frac{1}{p_{ij}} \mathbf{W}_m^k \mathbf{a}_j^{k-1} \Big), \tag{2}$$

where $1/p_{ij} = 1/\sqrt{\mathcal{N}_i| \times |\mathcal{N}_j|}$, $\mathbf{W}_m$ is the weight matrix, and σ is the activation function.

- **GAT** (graph attention networks [2]). In GAT, the Aggregate function is defined as:

$$\mathbf{a}_i^k = \|_{s=1}^S \sigma\Big(\sum\nolimits_{j\in\mathcal{N}_i\cup a_i} \alpha_{ij}^{k,s}\mathbf{W}_m^{k,s}\mathbf{a}_j^{k-1}\Big), \tag{3}$$

  where $\|$ denotes the concatenate operation and S denotes the number of attention heads. $\alpha_{ij}^{k,s}$ is the attention score between atoms a_i and a_j, which is defined as follows:

$$\alpha_{ij}^{k,s} = \frac{\sigma\Big(\mathbf{w}^{k,s\top}\big(\mathbf{W}_m^{k,s}\mathbf{a}_i^k\|\mathbf{W}_m^{k,s}\mathbf{a}_j^k\big)\Big)}{\sum_{j'\in\mathcal{N}_i\cup a_i}\sigma\Big(\mathbf{w}^{k,s\top}\big(\mathbf{W}_m^{k,s}\mathbf{a}_i^k\|\mathbf{W}_m^{k,s}\mathbf{a}_{j'}^k\big)\Big)}, \tag{4}$$

  where $\mathbf{w}$ is a learnable vector in the attention mechanism.

- **SAGE** (graph sample and aggregate [3]). We utilize the mean pooling variant of SAGE and its Aggregate function is defined as follows:

$$\mathbf{a}_i^k = \sigma\Big(\mathbf{W}_m^{k,1}\big(\mathbf{a}_i^{k-1}\|\frac{1}{|\mathcal{N}_i|}\sum\nolimits_{j\in\mathcal{N}_i}\mathbf{W}_m^{k,2}\mathbf{a}_j^{k-1}\big)\Big). \tag{5}$$

  In addition to the mean operation, max and LSTM pooling operations could also be used in the Aggregate function.

- **TAG** (topology adaptive graph neural networks [4]). We use $\mathbf{A}^k$ as the atom embedding matrix in layer k. In TAGCN, the Aggregate function is defined as:

$$\mathbf{A}^k = \sigma\Big(\sum\nolimits_{f=0}^{F}\tilde{P}\mathbf{A}^{k-1}\mathbf{W}^{k,l}\Big), \tag{6}$$

  where F is the local filter size, $\tilde{P} = D^{-1/2}PD^{-1/2}$ denotes the normalized adjacency matrix, P is the adjacency matrix, and D is the degree matrix.

B. Functional Group Information

In reaction-aware graph learning, we use functional group information as initial features for molecules. Specifically, we consider 39 functional groups from RDKit (an open-source chem-informatics software[1]), as shown in Table 1.

C. Molecule Embedding Analysis

C.1. Molecular Embedding Analysis of 39 Functional Groups

We explore the relationship between the visualized embeddings and the molecular functional group properties. Specifically, we conduct experiments based on the 39 functional groups

[1]https://www.rdkit.org/

Table 1: Summary of functional groups.

Symbol	Explanation	Symbol	Explanation
-NC(=O)CH3	methyl amide	-SO2Cl	sulfonyl chloride
-C(=O)O	carboxylic acids	-SOCH3	methyl sulfinyl
-C(=O)OMe	carbonyl methyl ester	-SCH3	methylthio
-C(=O)H	terminal aldehyde	-S	thiols
-C(=O)N	amide	=S	thiocarbonyls
-C(=O)CH3	carbonyl methyl	-SO2CH3	methyl sulfonyl
-N=C=O	isocyanate	-tBu	t-butyl
-N=C=S	isothiocyanate	-CF3	trifluoromethyl
-NO2	nitro	-C#CH	acetylenes
-N=O	nitroso	-cPropyl	cyclopropyl
=N-O	oximes	-X	halogens
=NCH3	imines	-OMe	methoxy
-N=CH2	imines	-O	side-chain hydroxyls
-N=NCH3	terminal azo	=O	side-chain aldehydes
-N=N	hydrazines	-N	primary amines
-N#N	diazo	=N	imine
-C#N	cyano	#N	nitriles
-SO2NH2	primary sulfonamide	-OEt	ethoxy
-NHSO2CH3	methyl sulfonamide	-SO3CH3	methyl ester sulfonyl
-SO3H	sulfonic acid		

listed in Table 1. These 39 functional groups reflect various aspects of molecular properties. Based on these functional groups, we conduct molecular visualized embedding experiments, as illustrated in Figure 1. In cases where a functional group is absent in all molecules within the dataset, the corresponding visualization figures are not plotted. From the figures, it can be observed that, in the two-dimensional space, the embeddings generated by our method show a correlation with some functional groups (e.g., '-O', '=O', and '-C(=O)N'), clustering molecules with these functional groups to some extent. For the majority of functional groups, due to their low occurrence in the dataset, it is challenging to observe a clear correlation between molecule embeddings and functional group properties.

C.2. Molecule Embedding Cluster Analysis

In this section, we further analyze the molecule embeddings through clustering. Specifically, we conduct clustering analysis using permeability, molecule size, and hydroxyl functional groups as examples. The results are shown in Figure 2. We use the k-means method to cluster the molecule embeddings into eight clusters. For each cluster, we calculate the percentage of molecules with different property labels relative to the total number of molecules in that cluster. For example, in cluster 3 shown in Figure 2(c), 81.2% of the molecules have the property label "$OH\ groups = 0$". Generally, we observe that each cluster has a representative property

label (i.e., a label with a high percentage). These results suggest that the learned molecule embeddings effectively group molecules with similar property labels together.

References

[1] Kipf T N, Welling M. Semi-supervised classification with graph convolutional networks[J]. arXiv preprint arXiv:1609.02907, 2016.

[2] Veličković P, Cucurull G, Casanova A, et al. Graph attention networks[J]. arXiv preprint arXiv:1710.10903, 2017.

[3] Hamilton W, Ying Z, Leskovec J. Inductive representation learning on large graphs[J]. Advances in neural information processing systems, 2017, 30.

[4] Du J, Zhang S, Wu G, et al. Topology adaptive graph convolutional networks[J]. arXiv preprint arXiv:1710.10370, 2017.

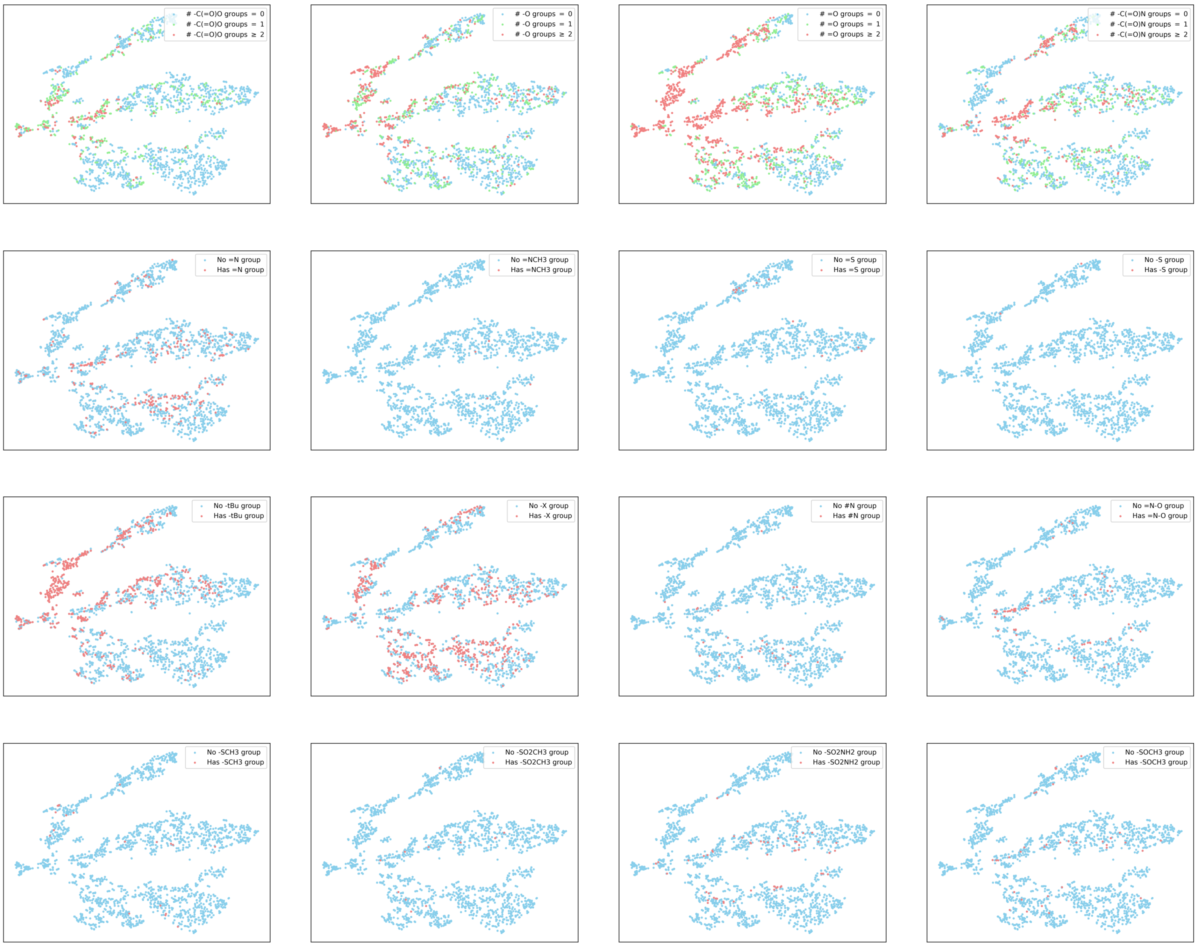

Figure 1 (continued on next page): Visualized molecule embedding under 39 molecular functional groups on the BBBP dataset. In cases where a functional group is absent in all molecules within the dataset, the corresponding visualization figures are not plotted.

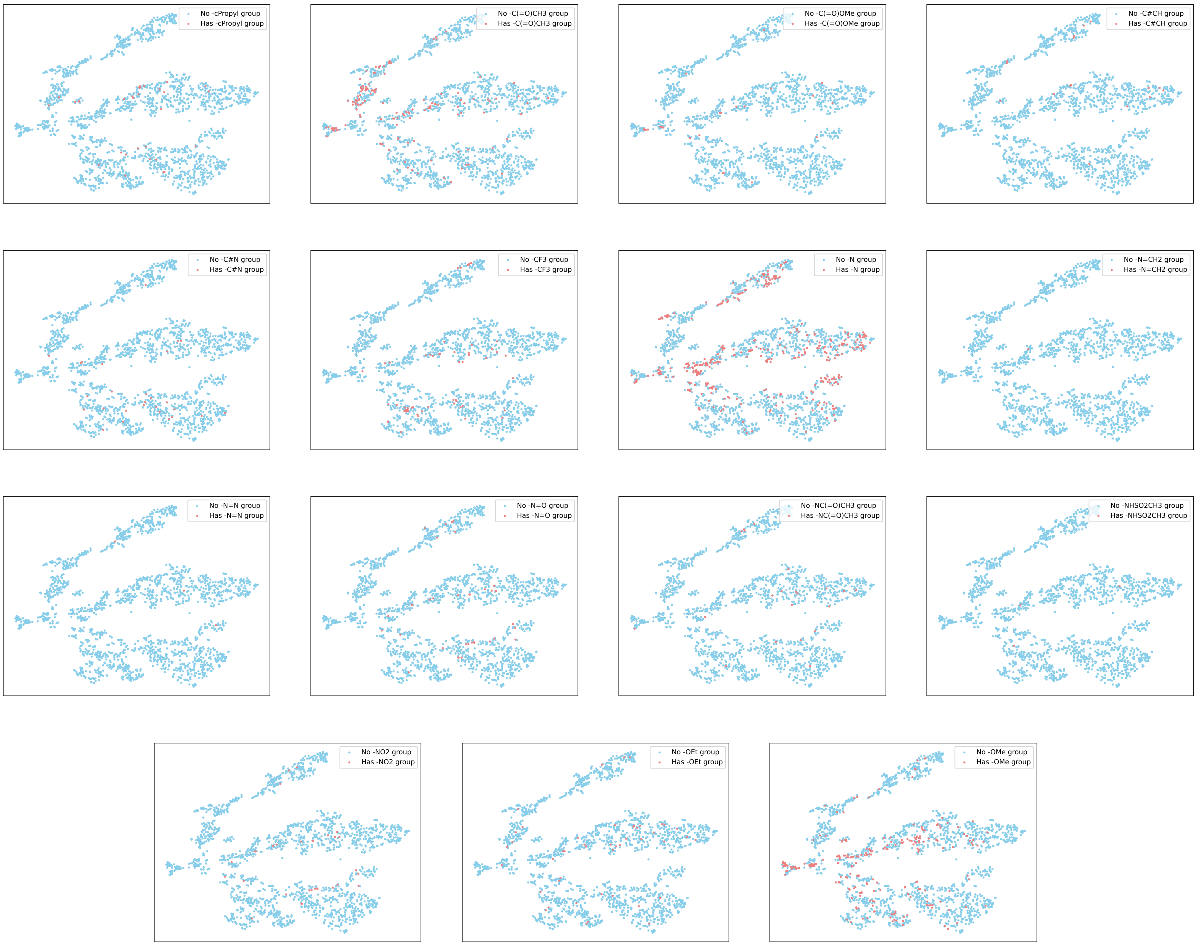

Figure 1 (continued): Visualized molecule embedding under 39 molecular functional groups on the BBBP dataset. In cases where a functional group is absent in all molecules within the dataset, the corresponding visualization figures are not plotted.

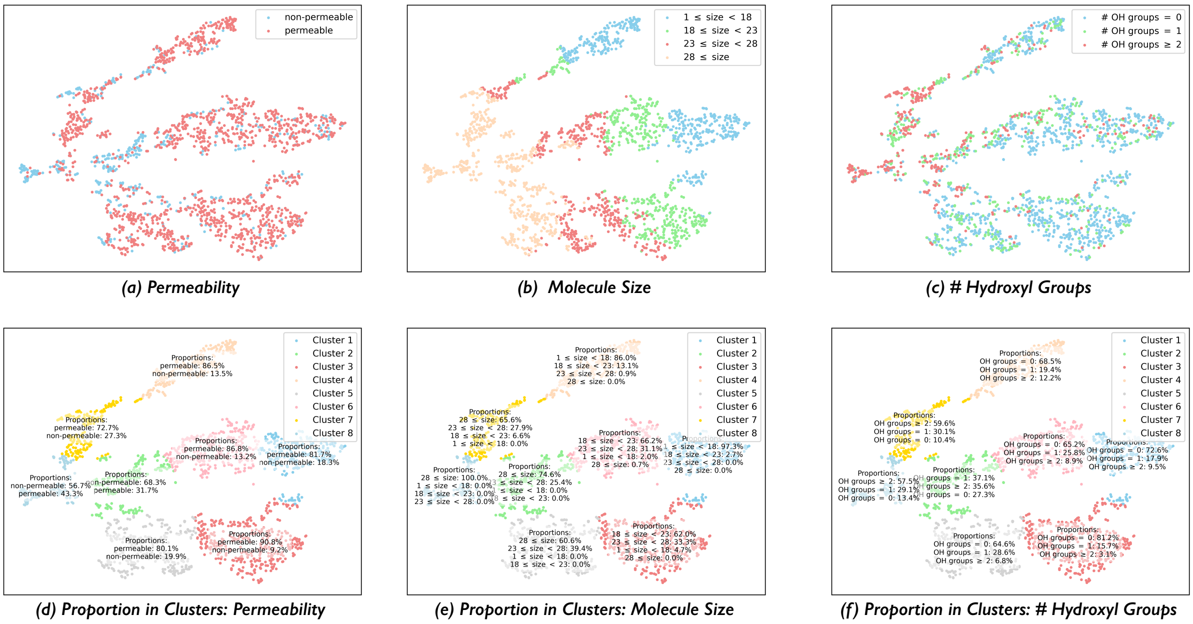

(a) Permeability *(b) Molecule Size* *(c) # Hydroxyl Groups*

(d) Proportion in Clusters: Permeability *(e) Proportion in Clusters: Molecule Size* *(f) Proportion in Clusters: # Hydroxyl Groups*

Figure 2: Molecule Embedding Cluster Analysis on the BBBP dataset.
